# Dietary Selenium Alleviated Mouse Liver Oxidative Stress and NAFLD Induced by Obesity by Regulating the KEAP1/NRF2 Pathway

**DOI:** 10.3390/antiox11020349

**Published:** 2022-02-10

**Authors:** Yi Wang, Bingbing Liu, Peixuan Wu, Yi Chu, Sisi Gui, Yazhen Zheng, Xiaodong Chen

**Affiliations:** 1 Key Laboratory of Agricultural Animal Genetics, Breeding and Reproduction of Ministry of Education, College of Animal Science and Technology & College of Veterinary Medicine, Huazhong Agricultural University, Wuhan 430070, China; wy@webmail.hzau.edu.cn (Y.W.); liubb@webmail.hzau.edu.cn (B.L.); cy313350648@webmail.hzau.edu.cn (Y.C.); gss.hzau.qq.com@webmail.hzau.edu.cn (S.G.); zhengyzhen@webmail.hzau.edu.cn (Y.Z.); 2 College of Life Science and Technology, Huazhong Agricultural University, Wuhan 430070, China; Wupeixuan@webmail.hzau.edu.cn

**Keywords:** NAFLD (nonalcoholic fatty liver disease), dietary selenium, oxidative stress, KEAP1/NRF2 pathway

## Abstract

Nonalcoholic fatty liver disease (NAFLD) occurs when excess fat is stored in the liver and it is strongly linked with metabolic syndrome and oxidative stress. Selenium (Se) is an essential micronutrient in animals, which has a variety of biological functions, including antioxidant and anti-inflammatory. However, the exact effect of dietary selenium on NAFLD and the underlying molecular mechanism are not yet clear. Herein, we fed a high-fat diet (HFD) to C57BL/6 mice to construct an in vivo NAFLD model, treated AML-12 cells with palmitic acid (PA) to construct an in vitro NAFLD model, and AML-12 cells were stimulated with H_2_O_2_ to induce hepatocyte oxidative stress and then treated with adequate selenium. We observed that adequate selenium significantly improved the hepatic injury and insulin resistance in HFD mice, and decreased the fat accumulation and the expression of lipogenic genes in PA-induced AML-12 cells. Meanwhile, selenium significantly inhibited the production of reactive oxygen species (ROS), inhibited apoptosis, and restored mitochondrial number and membrane potential in PA- induced AML-12 cells. In addition, selenium can promote selenoproteinP1 (SEPP1) synthesis to regulate the Kelch-like ECH-associated protein 1 (KEAP1)/NF-E2-related factor 2 (NRF2) pathway, so as to defend against hepatocyte oxidative stress. These findings suggest that dietary selenium supplementation can effectively resist hepatic injury and insulin resistance during NAFLD development, and regulate the KEAP1/NRF2 pathway to resist oxidative stress by promoting SEPP1 synthesis.

## 1. Introduction

In recent years, NAFLD has been considered to be one of the most common liver diseases worldwide, with about 30% of the population in the world being affected [1]. NAFLD refers to the excessive accumulation of triglycerides (TG) in liver cells without excessive alcohol stimulation or viral invasion, which is a general term for a series of diseases characterized by significant accumulation of liver lipids [2]. Obesity is the most important risk factor for the development of NAFLD. According to the epidemiological statistical analysis, the incidence of NAFLD in obese patients is 4.6 times higher than that in normal weight patients [2,3,4]. NAFLD not only causes common liver diseases, such as nonalcoholic steatohepatitis (NASH) and liver fibrosis, but also causes complications such as insulin resistance (IR), high blood pressure, dyslipidemia, type 2 diabetes (T2DM), and cardiovascular diseases, and eventually hepatocellular cancer (HCC) [3]. On the pathogenesis of NAFLD, a “two hit hypothesis” was proposed [4]. The “first hit” is characterized by liver fat accumulation, characterized by liver TG accumulation and insulin resistance, and sustained liver fat accumulation eventually leads to cirrhosis [2,4,5]. With the continuous development of NAFLD, the “second hit” appears, the most important of which is inflammatory cytokines, mitochondrial dysfunction, and oxidative stress. The second hit results in severe damage to liver function, and ultimately liver fibrosis [6,7]. This suggests that hepatic injury caused by NAFLD is closely related to oxidative stress. Oxidative stress is due to the dysfunction of the cellular antioxidant system induced by excessive production of ROS. Oxidative stress can lead to serious failure of cell function and eventually cell death [8]. ROS are highly active and unstable free radical compounds [9]. Organisms may also contain a certain amount of ROS in normal cell metabolism [8,9]. ROS has a physiological effect at a lower level, but a large amount of ROS produced by cells under certain stimuli can cause severe damage to cells [6]. ROS can damage DNA and change the expression of certain genes. They can also induce protein and lipid oxidation, which alters the functional activity of some enzymes, structural proteins, and cell membranes [10]. Oxidative stress generated during the development of NAFLD can cause great damage to hepatocytes, so the screening of drugs and food additives that can prevent NAFLD and relieve hepatocyte oxidative stress has always been a focus of NAFLD research. In this study, we selected selenium source food additives as the research object.

Selenium (Se) as an essential micronutrient in animals; it plays a vital role in several regulatory and metabolic functions of the body, such as antioxidant, anti-cancer, and detoxification [11,12]. In the liver, dietary selenium is metabolized to selenide, which is subsequently used for the synthesis of selenoproteins [13]. The major selenoproteins in the liver include SEPP1 and glutathione peroxidase1 (GPX1) [14]. SEPP1 is a glycoprotein rich in selenocysteine, synthesized and primarily secreted by hepatocytes, and functions as the main Se transporter from the liver to other tissues [12,14,15]. SEPP1 is closely related to the occurrence and development of NAFLD. Some studies have proved that SEPP1 can regulate the AMPK/ACC pathway to regulate the development of NAFLD [16]. A large number of studies on Se focus on antioxidation. Some studies have proved that Se plays an important role in NAFLD. However, whether Se has a lipid-lowering effect and the molecular mechanism of its resistance to oxidative stress in hepatocytes are still unclear.

In mammals, the NRF2-KEAP1 system is a defense system designed to protect the normal physiological state of cells [7]. The system is regulated by the interaction between NRF2 and the cell sol inhibitor KEAP1 [17]. A major emerging function of NRF2 is its role in resistance to oxidative stress. Some studies have shown that promoting NRF2 activity with some beneficial drugs can protect animals from oxidative damage [7,17,18]. Under normal circumstances, NRF2 and KEAP1 bind to each other in the cytoplasm and then degrade through the ubiquitous protein-prosthetic pathway in a way on which KEAP1 relies [19]. However, in the presence of ROS, NRF2 is isolated from KEAP1; the degradation is stopped; and then accumulates in the nucleus and becomes heterogeneous with small Musculoaponeurotic fibrosarcoma oncogene (Maf) family proteins, which can be activated by antioxidant reaction elements (ARE) to protect the target gene of cells [7,19]. Therefore, the level of NRF2 protein is regulated by the degradation process, and the stability of NRF2 is the key to the response of cells to oxidative stress.

In the present study, we established an NAFLD model in vivo and in vitro as well as hepatocyte oxidative stress model, and then treated with appropriate Se to evaluate the role of Se defense against NAFLD, the antioxidant effect, and its latent molecular mechanism. Our results demonstrate that Se can ameliorate hepatic injury induced by NAFLD and promote SEPP1 synthesis to regulate the KEAP1/NRF2 pathway, so as to defend against hepatocyte oxidative stress.

## 2. Materials and Methods

### 2.1. Materials

DMEM/F12 media, fetal bovine serum, and insulin-transferrin-selenium solution were purchased from Gibco (Beijing, China). SEPP1 antibody was purchased from Abcam (HKSP, N.T. Hong Kong, China). Nrf2 antibody was purchased from Abclonal (Wuhan, China). GPx1 antibody was purchased from Santa Cruz Biotechnology, Inc. (Santa Cruz, CA, USA). GAPDH antibody was purchased from Servicebio (Wuhan, China). PGC-1α antibody, HIS3 antibody, and NQO1 antibody were purchased from Proteintech Group, Inc. (Wuhan, China). Mito Tracker green, Reactive Oxygen Species Assay Kit, and Cell Counting Kit-8 were purchased from Beyotime (Shanghai, China). JC-1 mitochondrial staining kit was purchased from Biosharp (Shanghai, China). Annexin V-FITC/PI Apoptosis Detection kit was purchased from YEASEN (Shanghai, China). Sodium selenite (Na_2_SeO_3_) was purchased from sigma (Shanghai, China). L-Se-methylselenocysteine (L-SeMC) was purchased from Macklin (Shanghai, China). Palmitic acid (PA) was purchased from sigma (Shanghai, China). Dexamethasone was purchased from Solarbio (Beijing, China). Selenium-enriched Spirulina (Se content 350 mg/kg) was provided by Professor Kaiyao Huang from the Institute of Hydrobiology, Chinese Academy of Sciences (Wuhan, China). Hematoxylin and Eosin Staining Kit was purchased from Beyotime (Shanghai, China). Masson Stain Kit was purchased from YEASEN (Shanghai, China).

### 2.2. Animal Studies

C57BL/6 mice (25 g), 8-week-old males, bred by our laboratory. Animals were housed in cages in a room with a 12:12 h light/dark cycle set at an ambient temperature (22–25 °C). A total of 24 mice were randomly categorized into four groups with 6 mice in each group and fed for 12 weeks as follows: normal diet (CON, Se content 0.1 mg/kg, *n* = 6), ND with Se-enriched spirulina (Se, Se content 0.45 mg/kg, *n* = 6), high-fat diet-induced obese mice (HFD, 60% of calories from fat, research diets, *n* = 6), and HFD with Se-enriched spirulina (HFD + Se, Se content 0.45 mg/kg, *n* = 6). Se-enriched spirulina was supplied in a mixed chow diet. The food intake and body weight of each mouse were recorded weekly. After 12 weeks, mice were anesthetized by injection of 1% pentobarbital sodium (50 mg/kg), and blood samples were taken from the heart. Mice were then sacrificed with CO_2_ to take liver tissue samples. The serum and tissue samples were stored at −80 °C for further studies. All the animal procedures were approved by the Animal Ethical and Welfare Committee, Huazhong Agricultural University, and Hubei Province Committee on Laboratory Animal Care, P.R. China (protocol code: HZAUMO-2022-0013; date of approval: 8 January 2021).

### 2.3. Serum Analysis

Serum AST, ALT, TC, and TG levels were measured through Baiqiandu Biotechnology Co., Ltd. (Wuhan, China).

### 2.4. Glucose Tolerance Test

The intraperitoneal glucose tolerance test (IPGTT) was performed after mice were fasted overnight for 8 h. Venous blood was then collected from the tails and subjected to glucose measurements at 0 min (baseline) and then at 15, 30, 60, 90, and 120 min after intraperitoneal injection of glucose (1 g/kg body weight).

### 2.5. H&E Staining

H&E staining was carried out strictly according to the instructions of the Hematoxylin and Eosin Staining Kit. The liver specimens, fixed in 4% neutral-buffered paraformaldehyde for one day, were dehydrated and embedded in paraffin, and then sliced into 5 μm sections. Afterwards, the liver sections were dewaxed and stained with hematoxylin for 5–10 min, and then counterstained with eosin for 1–2 min. After washing two times with 70% ethyl alcohol, the images were captured under an inverted microscope (Olympus Corporation, Tokyo, Japan) at 400× magnification.

### 2.6. Masson Staining

Masson staining was carried out strictly according to the instructions of the Masson Stain Kit. The liver specimens, fixed in 4% neutral-buffered paraformaldehyde for one day, were dehydrated and embedded in paraffin, and then sliced into 5 μm sections. Afterwards, the liver sections were dewaxed and stained with hematoxylin for 60 s, and then counterstained with acid fuchsin pulp dying solution for 30–60 s; phosphomolybdic acid color separation solution for color separation for 6–8 min; and finally, aniline blue re-dyeing solution was added for dyeing for about 5 min, and it was discarded and rinsed with absolute ethanol. The images were captured under an inverted microscope (Olympus Corporation) at 400× magnification.

### 2.7. Cell Culture

AML12 (alpha mouse liver 12) cells are hepatocytes isolated from the normal liver of a 3-month-old mouse. Aml-12 cell line used in this study was purchased from Procell (Wuhan, China). AML-12 cells were maintained in DMEM/F12 with 10% feta bovine serum, 1% insulin-transferrin-selenium solution, and 40 ng/mL dexamethasone, and were incubated at 37 °C in 5% CO_2_. Formal experiments were carried out using cells from five generations later. AML-12 cells were inoculated in a well plate for culture and treated with PA when 80% to 90% fusion was achieved.

### 2.8. Quantitative Real Time PCR (qPCR)

A total of 2 μg RNA of each sample was used to perform reverse transcription. qPCR was carried out in a total volume of 20 μL with SYBR-Green mix (TransGen, Beijing, China) on an IQ5 thermal cycler (Bio-Rad, Hercules, CA). Musculus β-actin was used as the internal references and amplified in parallel. The PCR conditions were 95 °C for 3 min, followed by 40 cycles of 95 °C for 15 s, 60 °C for 15 s, and 72 °C for 15 s. Cycle threshold values were normalized to that of the internal references, and the relative gene expression levels were calculated by the 2^−^ΔΔCt^ method. The primer sequences are mostly derived from Primer Bank [20] and are shown in Table 1.

### 2.9. Western Blot

Proteins were extracted by RIPA buffer. Aliquots containing 60 μg of protein from each sample were separated by 10% SDS–PAGE, then all proteins were transferred onto polyvinylidene difluoride membranes. The membranes were blocked with TBST buffer (20 mM Tris–HCl, 137 mM NaCl, and 0.05% Tween-20) containing nonfat dried milk at room temperature for 60 min, and then incubated overnight with a 1:1000 dilution of appropriate primary antibodies. After extensive washing, the membranes were incubated with horseradish peroxidase-conjugated secondary antibodies (Servicebio, 1:10,000) for 1 h at room temperature and visualized using an ECL Western blotting detection system (Tiangen, Beijing, China).

### 2.10. Annexin V/PI Apoptosis Staining

Apoptosis was detected by Annexin V/PI apoptosis staining kit. AML-12 cells were fixed in 4% neutral-buffered paraformaldehyde for 15 min. After cleaning with PBS, 100 μL 1× Binding Buffer, 5 μL Annexin V-FITC, and 10 μL PI Staining Solution were added, it was kept away from light and reacted at room temperature for 10–15 min, and then 400 μL 1× Binding Buffer was added. Images were obtained by Laser confocal microscope (Carl Zeiss, Oberkochen, Germany). The Annexin V-FITC fluorescence signal is green and PI fluorescence signal is red. Early apoptotic cells had only strong green fluorescence, and late apoptotic cells had double staining of green and red fluorescence.

### 2.11. Mito Tracker

Mitochondria of AML-12 cells was detected by Mito-tracker staining kit. According to the experimental protocol, Mito-Tracker Green with a final concentration of 1 mM was prepared with anhydrous dimethyl sulfoxide (DMSO) as a stock solution, and diluted with DMEM medium to a working concentration of 50 nM [21]. Green fluorescence image was obtained by laser confocal microscope (Carl Zeiss).

### 2.12. JC-1 Staining

The mitochondrial membrane potential of AML-12 cells was detected by JC-1 staining kit. AML-12 cells were fixed in 4% neutral-buffered paraformaldehyde for 15 min. JC-1 dyeing working solution was added and mixed well. The cells were incubated at 37 °C for 20 min. After incubation at 37 °C, the working solution was sucked out and stained with JC-1 buffer (1×), and then washed twice. Image was obtained by laser confocal microscope (Carl Zeiss).

### 2.13. Cell Viability Assay

Cell viability of AML-12 was tested by Cell Counting Kit (CCK-8) kit. According to the measurement protocol, 10 ul enhanced CCK-8 solution was to the cell plate for 4 h, and the absorbance was measured at 450 nm using a microplate reader (Bio-Tek, Burlington, VT, USA).

### 2.14. Oil Red O Staining

Briefly, cells were washed twice with PBS, fixed in 4% paraformaldehyde for 0.5 h, and stained for 30 min with a 0.5% Oil Red O solution in 60% isopropanol. The cells were washed with PBS before analysis [22]. The images were captured under an inverted microscope (Olympus Corporation) at 400× magnification

### 2.15. Immunofluorescence

The method was performed according to the previous publication [23]. Cells on a sheet of glass were fixed with 4% paraformaldehyde (PFA) for 30 min and then washed twice with PBS. Next, cells were permeated with PBS containing 0.1% Triton X-100 for 5 min. The cells were incubated with 5% skim milk powder to block non-specific staining. After 12 h of incubation with primary antibody at 4 °C, the cells were washed three times with PBS. Later on, the samples were incubated with 10% goat serum at room temperature for 1 h. Then, cells were further stained with fluorophore (Alex488)-conjugated secondary antibodies. After staining, the cells were counterstained with DAPI.

### 2.16. Statistical Analysis

For in vivo experiments, six mice were used in each group. In vitro experiments were performed at least three times with similar results. Data were expressed as the means ± SEM. Statistical analysis was performed using GraphPad Prism 8.2 (GraphPad Software, Inc., San Diego, CA, USA). One-way-analysis of variance (ANOVA) and two-way-ANOVA with Tukey’s multiple comparisons tests were conducted. An adjusted *p*-value of <0.05 was considered as statistical significance.

## 3. Results

### 3.1. Effect of Dietary Selenium on Hepatic Injury and Insulin Resistance In Vivo Models of NAFLD

**Figure 1:**
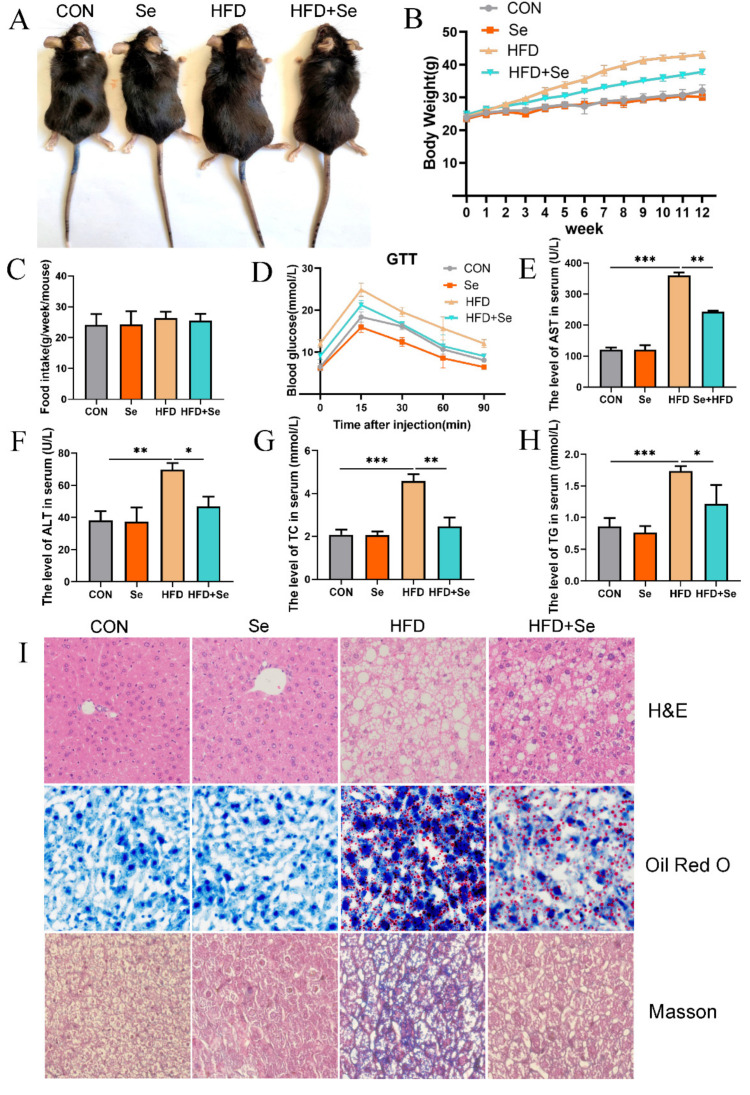
Effect of dietary selenium on hepatic injury and insulin resistance in HFD-induced NAFLD mice. (**A**) The appearances of C57BL/6 J mice in different groups were photographed in the last week. (**B**) The body weights of mice were monitored for 12 weeks. (**C**) Food intake was monitored every week. (**D**) The results of IPGTT. (**E**) The levels of serum AST (*n* = 6, *** *p* < 0.001, ** *p* < 0.01). (**F**) The levels of serum ALT (*n* = 6, ** *p* < 0.01, * *p* < 0.05). (**G**) The levels of serum TC (*n* = 6, ** *p* < 0.01, *** *p* < 0.001). (**H**) The levels of serum TG (*n* = 6, * *p* < 0.05, *** *p* < 0.001). (**I**) H&E staining, Oil Red O staining, and Masson staining of liver section (400× magnification).

During the 12-week period, Se-enriched spirulina was supplied to HFD. Visual observation showed that body weight was remarkably reduced in the Se + HFD group compared with the HFD group. Moreover, there was no significant difference in body weight between the Se group and control group (Figure 1A). During feeding, the weight of the HFD group increased rapidly, and adequate Se could inhibit the weight gain induced by HFD (Figure 1B). Meanwhile, the results also showed that adequate Se changed the body weight of HFD mice, but did not affect the food intake (Figure 1C). According to the results of IPGTT, HFD significantly impaired glucose tolerance in mice, and adequate Se reversed this effect (Figure 1D). ALT and AST are the canonical markers of liver injury. The levels of serum ALT and AST in HFD mice were significantly increased compared with those of in control mice, whereas the changes were markedly reversed by adequate Se (Figure 1E,F). In addition, through detecting the lipid profiles in serum, it was found that adequate Se could significantly inhibit the increase in serum TC and TG in HFD mice (Figure 1G,H). Meanwhile, H&E staining and oil red O staining showed that adequate Se significantly inhibited liver lipid accumulation in HFD mice. Moreover, Masson staining showed that adequate Se inhibited liver fibrosis in HFD mice (Figure 1I).

### 3.2. Selection of the Best Therapeutic Concentration of Na_2_SeO_3_ and L-SeMC in Palmitic Acid (PA)-Induced AML-12 Hepatocytes

**Figure 2:**
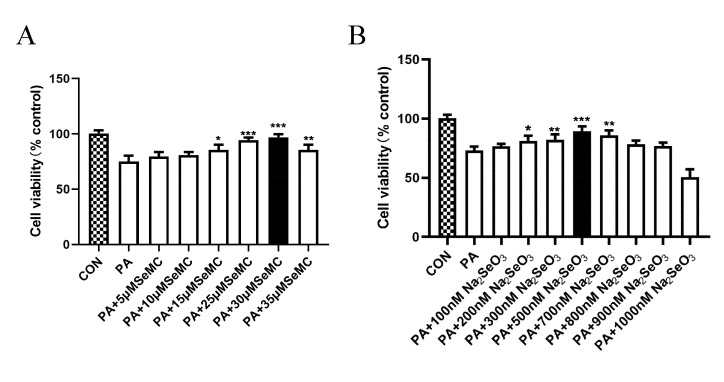
Selection of the best therapeutic concentration of Na_2_SeO_3_ and L-SeMC in PA-induced AML-12 cells. (**A**) Cell viability of AML-12 cells was measured by CCK-8 after co-treatment with 300 μM PA and different concentrations of L-SeMC (0–35 μM) for 24 h (*n* = 7, *** *p* < 0.001, ** *p* < 0.01, * *p* < 0.05 vs. PA group). (**B**) Cell viability of AML-12 cells was measured by CCK-8 after co-treatment with 300 μM PA and different concentrations of Na_2_SeO_3_ (0–900 nM) for 24 h (*n* = 7, *** *p* < 0.001, ** *p* < 0.01, * *p* < 0.05 vs. PA group).

In this study, palmitic acid (PA) was used to induce mice-derived AML-12 hepatocytes and establish an in vitro NAFLD model, and Na_2_SeO_3_ and L-SeMC as a Se source were supplemented in cell culture medium. CCK-8 kit was used to determine the best beneficial concentration of Na_2_SeO_3_ and L-SeMC in PA-induced AML-12 cells. When AML-12 cells were treated with Na_2_SeO_3_ or L-SeMC alone, it was found that Na_2_SeO_3_ and L-SeMC could increase cell viability of AML-12 cells, and among which 500 nM Na_2_SeO_3_ and 30 μM L-SeMC had the best therapeutic effect (Supplemental Figure S1A,B). Moreover, the result showed that 300 μM PA can significantly inhibit cell viability without causing a large number of cell deaths (Supplemental Figure S1C). Furthermore, Na_2_SeO_3_ and L-SeMC could significantly protect against 300 μM PA-induced decreased cell viability, of which 500 nM Na_2_SeO_3_ and 30 μM L-SeMC had the best therapeutic effect (Figure 2A,B). In summary, 500 nM Na_2_SeO_3_, 30 μM L-SeMC, and 300 μM PA were used in the following experiment to further verify the protective efficacy of Se and explore its underlying mechanisms.

### 3.3. Effects of Selenium on Lipid Accumulation In Vitro Models of NAFLD

**Figure 3:**
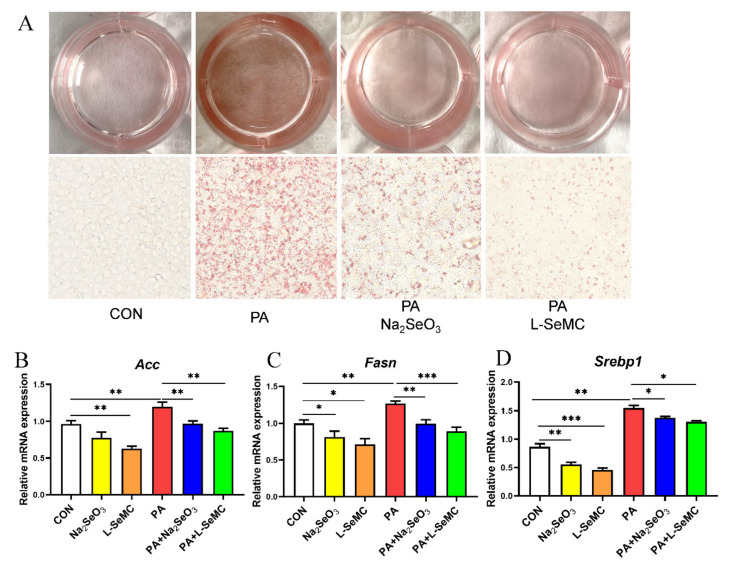
Effects of Na_2_SeO_3_ and L-SeMC on lipid accumulation in PA-induced AML-12 cells. (**A**) Oil Red O assay was used to measure lipid accumulation in AML-12 cells after co-treatment with PA with or without Na_2_SeO_3_ and L-SeMC for 24 h (400× magnification). (**B**–**D**) The mRNA levels of *Acc*, *Fasn*, and *Srebp1* (*n* = 3, *** *p* < 0.001, ** *p* < 0.01, * *p* < 0.05).

AML-12 cells were treated with 300 μM PA, 500 nM Na_2_SeO_3_ + 300 μM PA, and 30 μM L-SeMC + 300 μM PA for 24 h, respectively, and then detected with Oil Red O staining. The results showed that PA significantly increased lipid accumulation in AML-12 cells, which was markedly alleviated by Na_2_SeO_3_ and L-SeMC (Figure 3A). PA significantly increased the mRNA levels of *Acc*, *Fasn*, and *Srebp1*, while Na_2_SeO_3_ and L-SeMC significantly decreased the mRNA levels of *Acc*, *Fasn*, and *Srebp1* in PA-induced AML-12 cells. Moreover, when AML-12 cells were only treated with Na_2_SeO_3_ or L-SeMC, the mRNA levels of *Acc*, *Fasn*, and *Srebp1* were also decreased (Figure 3B–D).

### 3.4. Effects of Selenium on Oxidative Stress in In Vitro Models of NAFLD

**Figure 4:**
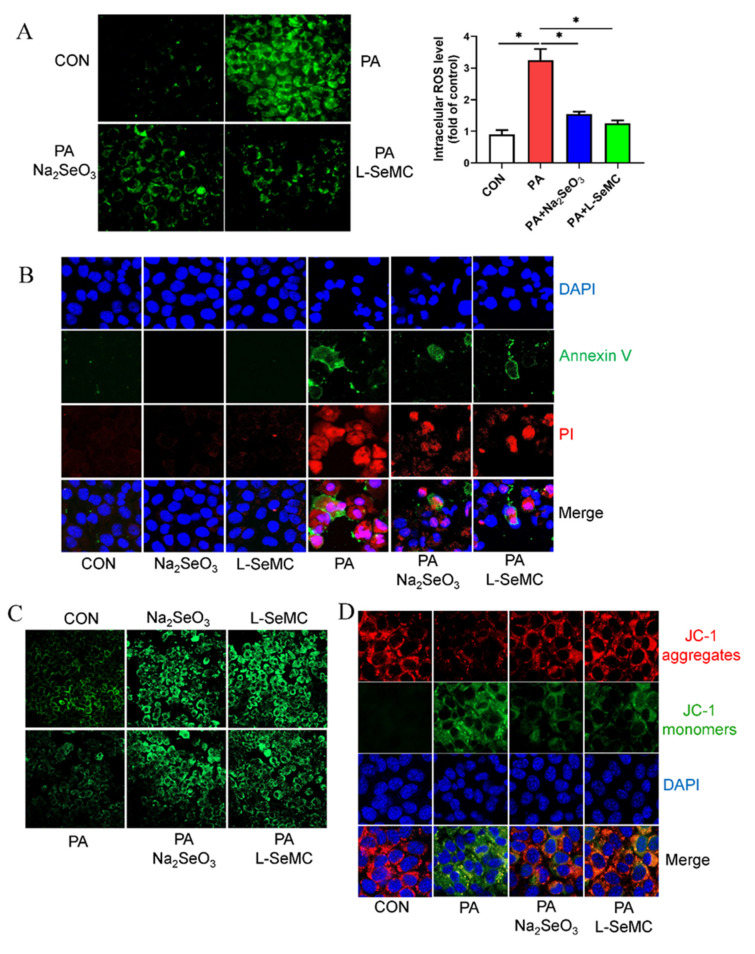
Effects of Na_2_SeO_3_ and L-SeMC on oxidative stress in PA-induced AML-12 cells. (**A**) ROS content detection in AML-12 cells after co-treatment with PA with or without Na_2_SeO_3_ and L-SeMC for 24 h, including images (400× magnification) and fluorescence density analysis (*n* = 3, * *p* < 0.05). (**B**) The apoptosis of AML-12 cells was determined by Annexin V/PI apoptosis staining kit after co-treatment with PA with or without Na_2_SeO_3_ and L-SeMC for 24 h (200× magnification). (**C**) Mitochondrial content was measured by Mito traker green after co-treatment with PA with or without Na_2_SeO_3_ and L-SeMC for 24 h (400× magnification). (**D**) Mitochondrial membrane potential was measured by JC-1 staining kit after co-treatment with PA with or without Na_2_SeO_3_ and L-SeMC for 24 h (400× magnification).

AML-12 cells were treated with 300 μM PA, 500 nM Na_2_SeO_3_ + 300 μM PA, and 30 μM L-SeMC + 300 μM PA for 24 h, respectively, and then the production of ROS was detected. The results showed that a large number of ROS were produced in PA-induced AML-12 cells, but Na_2_SeO_3_ and L-SeMC could significantly protect against such changes (Figure 4A). In addition, the results of Annexin V/PI apoptosis staining showed that cells induced strong apoptosis fluorescent signals in PA-induced AML-12 cells, but Na_2_SeO_3_ and L-SeMC significantly reduced fluorescent signals. Meanwhile, the results showed that only the use of Na_2_SeO_3_ or L-SeMC to treat AML-12 cells did not lead to apoptosis (Figure 4B). The results of Mito Tracker staining showed that the number of mitochondria in PA-induced AML-12 cells decreased, while Na_2_SeO_3_ and L-SeMC could reverse the effect of PA. Moreover, in the absence of PA, Na_2_SeO_3_ and L-SeMC still increased the mitochondrial content of AML-12 cells (Figure 4C). JC-1 staining is used to test the mitochondrial membrane potential. The results showed that mitochondrial membrane potential decreases in PA-induced AML-12 cells. However, Na_2_SeO_3_ and L-SeMC restored the mitochondrial membrane potential to normal in PA-induced AML-12 cells (Figure 4D).

### 3.5. Effects of Selenium on the KEAP1/NRF2 Pathway in the In Vitro Models of NAFLD

**Figure 5:**
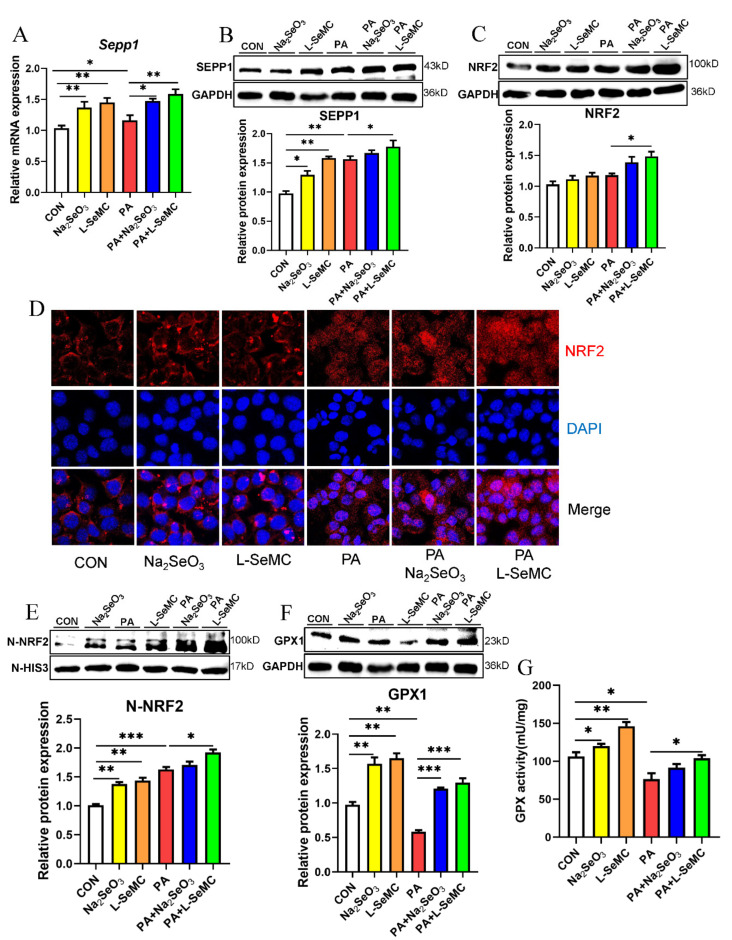
Effects of Na_2_SeO_3_ and L-SeMC on the Keap1/Nrf2 pathway in PA-induced AML-12 cells. (**A**) The mRNA level of *Sepp1* (*n* = 3, ** *p* < 0.01, * *p* < 0.05). (**B**) The protein level of SEPP1 (*n* = 3, ** *p* < 0.01, * *p* < 0.05). (**C**) The total protein level of NRF2 (*n* = 3, * *p* < 0.05). (**D**) The accumulation of NRF2 in the nucleus was detected by immunofluorescence (400× magnification). (**E**) The nuclear protein level of NRF2 (*n* = 3, *** *p* < 0.001, ** *p* < 0.01, * *p* < 0.05). (**F**) The protein level of GPX1 (*n* = 3, *** *p* < 0.001, ** *p* < 0.01). (**G**) The enzyme activity of GPX1 (*n* = 3, ** *p* < 0.01, *p < 0.05).

SEPP1 is the most important selenoprotein in the liver. The results of qPCR and Western blot showed that the mRNA and protein levels of SEPP1 increased in PA-induced AML-12 hepatocytes. Moreover, Na_2_SeO_3_ and L-SeMC further increased the mRNA and protein levels of SEPP1 (Figure 5A,B). The expression level of NRF2, a key antioxidant protein, was detected. The results showed that the total protein level of NRF2 increased and then further increased by Na_2_SeO_3_ and L-SeMC in PA-induced AML-12 cells. Meanwhile, it can be noted that the change trend of total protein level of NRF2 is similar to that of SEPP1 (Figure 5C). Immunofluorescence was used to detect NRF2 in the nucleus. The results illustrated that a large amount of NRF2 accumulated in the nucleus, and Na_2_SeO_3_ and L-SeMC promoted NRF2 accumulation in PA-induced AML-12 cells. Moreover, the results showed that, even without PA, Na_2_SeO_3_ and L-SeMC could make a small amount of NRF2 enter the nucleus (Figure 5D). The same result was obtained after extracting nuclear protein for Western blot detection (Figure 5E). The change trend of NRF2 protein level in nucleus is similar to that of SEPP1 protein level. By detecting the expression level of GPX1 of NRF2 downstream, it was found that the protein levels of GPX1 were decreased significantly in PA-induced AML-12 cells. However, Na_2_SeO_3_ and L-SeMC restored the protein level of GPX1 in PA-induced AML-12 cells (Figure 5F). As GPX1 is an antioxidant enzyme, it is necessary to detect the enzyme activity of GPX1. The results showed that the enzyme activity of GPX1 decreased significantly in PA-induced AML-12 cells, but Na_2_SeO_3_ and L-SeMC reversed this effect (Figure 5G).

### 3.6. Effects of Selenium on Oxidative Stress in H_2_O_2_-Induced AML-12 Hepatocytes

**Figure 6:**
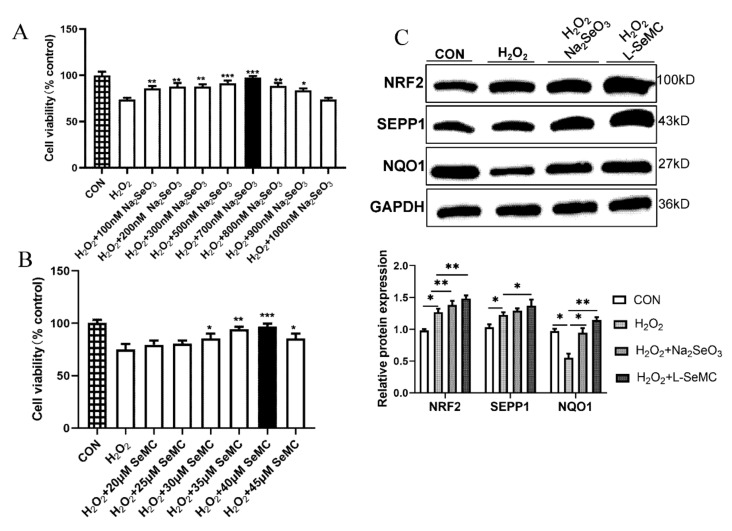
Effects of Na_2_SeO_3_ and L-SeMC on oxidative stress in H_2_O_2_-induced AML-12 cells. (**A**) Cell viability of AML-12 cells was measured by CCK-8 after co-treatment with 100 μM H_2_O_2_ and different concentrations of Na_2_SeO_3_ (0–1000 nM) for 3 h (*n* = 7, *** *p* < 0.001, ** *p* < 0.01, * *p* < 0.05 vs. H_2_O_2_ group). (**B**) Cell viability of AML-12 cells was measured by CCK-8 after co-treatment with 100 μM H_2_O_2_ and different concentrations of L-SeMC (0–45 μM) for 3 h (*n* = 7, *** *p* < 0.001, ** *p* < 0.01, * *p* < 0.05 vs. H_2_O_2_ group). (**C**) The protein level of SEPP1, NRF2, and NQO1 (*n* = 7, ** *p* < 0.01, * *p* < 0.05).

H_2_O_2_ is one of the endogenous ROS [13]. Therefore, H_2_O_2_ is a common oxidative stress inducer. H_2_O_2_ with different concentration gradients (0–800 μM) was used to treat AML-12 cells for 3 h, 6 h, and 9 h, respectively. The results of CCK-8 assay showed that H_2_O_2_ significantly reduced the cell viability of AML-12 cells. After treatment with 100 μM H_2_O_2_ for 3 h, the cell viability of AML-12 cells was decreased significantly (Supplemental Figure S2). However, Na_2_SeO_3_ and L-SeMC reversed the decrease in H_2_O_2_-induced AML-12 cell viability, of which 700 nM Na_2_SeO_3_ and 40 μM L-SeMC had the best effect (Figure 6A, B). Thus, in the following experiment, 700 nM Na_2_SeO_3_ and 40 μM L-SeMC were used in 100 μM H_2_O_2_-induced AML-12 cells for 3 h. The results of Western blot showed that the protein level of SEPP1 was increased in H_2_O_2_-induced AML-12 cells, and Na_2_SeO_3_ and L-SeMC further increased the protein level of SEPP1. Meanwhile, the change trend of NRF2 protein level was the same as that of SEPP1 (Figure 6C). NQO1 is a key enzyme regulating oxidative stress and an important downstream protein of NRF2 [9]. The results of Western blot show that H_2_O_2_ reduces the protein level of NQO1. This shows that the cellular antioxidant system is dysregulated. However, similar to the performance in PA-induced AML-12 cells, Na_2_SeO_3_ and L-SeMC reversed the effect of H_2_O_2_ (Figure 6C). These results suggested that adequate and appropriate Se could rescue the cell viability of H_2_O_2_-induced AML-12 cells, and the antioxidant mechanism of Se in H_2_O_2_-induced AML-12 cells is the same way as that in PA-induced AML-12 cells.

## 4. Discussion

The present study demonstrated that dietary selenium could reverse liver damage and insulin resistance in the progression of NAFLD, and alleviated oxidative stress in hepatocytes through the KEAP1/NRF2 pathway. In HFD-induced NAFLD mice, significant weight gain, insulin resistance, impaired hepatic function, hepatic steatosis, and hepatic fibrosis were observed, which were reversed by appropriate Se supplementation. More importantly, appropriate Se supplement alleviated hepatocyte oxidative stress by regulating the KEAP1/NRF2 pathway. Moreover, under normal conditions, appropriate Se supplement did not cause additional damage to the liver.

As a threat to human health, NAFLD has always been the focus of the world for its pathogenesis and prevention. For its pathogenesis, the theory of the “two hit hypothesis” has been widely recognized. According to the “two hit hypothesis” [4]. The “first hit”, namely steatosis, is the continuous accumulation of lipids in the liver caused by obesity and insulin resistance [24]. In the present study, we found HFD-induced NAFLD mice showed significant weight gain accompanied by serious hepatic steatosis and impaired hepatic function. However, according to the results of in vivo experiments, the weight gain of Se-adequate HFD mice was significantly inhibited compared with HFD mice (Figure 1A,B).

In addition, Se-adequate HFD mice significantly relieved liver steatosis and hepatic function was restored (Figure 1E–I). Some studies have shown that Se supplements, alone or in combination with other drugs, have a favorable effect on hepatic steatosis. Selenium-enriched Ziyang green tea can reduce oxidative stress and liver steatosis in high-fructose fed mice [25]. Co-administration of Se and magnesium can prevent the increase in blood lipid and liver function caused by HFD in rats; Se combined with zinc can improve lipid profile, liver function, and hepatic steatosis in rats [26]. Similarly, selenite alone or in combination with probiotics can reverse the adverse effects of HFD on lipid, liver function, and liver steatosis in albino mice [27]. In chickens, nano-elemental Se supplementation alleviates the abnormal fatty acid metabolism induced by hexavalent chromium by up-regulating acyl-CoA oxidase-1 and down-regulating fatty acid synthase-1, thus promoting fatty acid oxidation in liver and reducing de novo fat formation [28]. Similar results showed in our study that appropriate and adequate Se can reduce lipid accumulation by reducing the mRNA level of lipogenic genes in PA-induced AML-12 cells (Figure 3).

In addition, we found that Se significantly alleviated HFD-induced insulin resistance (Figure 1D). There have been reports that Se may have insulin-like effects by increasing tyrosine phosphorylation of insulin receptor β subunit and insulin receptor substrate-1 in rat primary hepatocytes and 3T3-L1 cells. Selenium has also been shown to activate mitogen-activated protein kinase (MAPK) signaling, which is involved in insulin signaling [29]. Selenium supplementation seems to have an important effect on liver fibrosis [30]. Se supplementation reduced the number of CCL4-induced hepatic stellate cells (HSCs) in mice, a key factor in the pathogenesis of liver fibrosis [31,32]. In other studies, Se and vitamin E combined administration also reduced liver fibrosis and promoted apoptosis of activated HSCs in CCL4-induced rat hepatic injury [33,34]. In this study, we also found that Se supplements can reverse HFD-induced liver fibrosis in mice, which may be based on the same mechanism (Figure 1I). Together, Se supplements can effectively alleviate the weight gain and insulin resistance induced by HFD, inhibit liver lipid accumulation and fibrosis, and exert a hepatoprotective effect in HFD mice. Moreover, Se supplements can down-regulate the expression level of lipogenic genes in hepatocytes and inhibit the accumulation of liver lipids. Therefore, adequate Se protects against the “first hit” in NAFLD. It can fundamentally inhibit the development of NAFLD.

As NAFLD continues to develop, the “second hit” occurs. The beginning of the “second hit” is based on steatosis. It further deepens the liver damage through the oxidative stress and inflammatory response induced by lipid peroxidation, causes mitochondrial dysfunction, and gradually develops NAFLD into NASH [4,35]. One of the most important characteristics of cell oxidative stress is the massive production of ROS in cells [8]. Studies have shown that Se can prevent cell apoptosis induced by ROS [36]. According to our results of the in vitro NAFLD model, adequate Se reversed the large production of ROS and apoptosis in PA-induced AML-12 cells (Figure 4A,B). Mitochondria is the main place to produce ROS. During oxidative stress, the normal function of mitochondria will be seriously damaged [37]. Interestingly, Se nanoparticles may induce mitochondrial dysfunction in cancer cells through the mitochondrial pathway, leading to apoptosis of hepatoma cell line HepG2, which confirms the anticancer effect of Se [38]. However, according to our results, Se has a protective effect on mitochondria in normal hepatocyte, and it can restore the mitochondrial number and membrane potential in PA-induced AML-12 cells (Figure 4C,D). On the whole, these exhibit the potential of defending against the “second hit” in NAFLD.

The role of Se depends on selenoproteins with multiple functions. The role of Se in liver mainly depends on SEPP1. SEPP1 is the most important selenoprotein in the liver. Few studies have linked SEPP1 to NAFLD. In lipopolysaccharide (LPS)-induced acute inflammatory response, the liver expression level of SEPP1 decreases, as does the blood Se level, which means that the liver SEPP1 expression is impaired during the acute phase of the response, thus affecting the transport and supply of Se to peripheral tissues [39]. In contrast, with hepatic steatosis and oxidative stress, HFD mice had a higher hepatic SEPP1 expression; this change may represent an offsetting mechanism against steatosis, oxidative stress, and HFD-induced chronic inflammation [40]. In this study, the expression level of SEPP1 increased in PA-induced AML-12 cells and H_2_O_2_-induced AML-12 cells. Moreover, Se supplements further increased the expression level of SEPP1 (Figure 5A,B). Meanwhile, we found that the change trend of NRF2 total protein level was the same as that of SEEP1 in PA-induced AML-12 cells (Figure 5B,C) and in H_2_O_2_-induced AML-12 cells (Figure 6C). Importantly, SEPP1 silencing reduces GPX1 activity [41]. GPX1 is also an important selenoprotein in the liver. It is an enzyme with a key antioxidant effect [42,43]. In addition, GPX1 is a downstream target protein of NRF2 [44]. Therefore, it can be speculated that the antioxidant mechanism of SEPP1 is related to NRF2.

NRF2 is an important nuclear transcription factor, which can promote the transcription of downstream antioxidant proteins, including NQO1, GPX1, and other important antioxidant enzymes, to regulate oxidative stress [45]. NRF2 is closely related to the function of mitochondria [46]. At present, research has proved that melatonin can ameliorate trimethyltin chloride-induced cardiotoxicity through KEAP1/NRF2, and that Cimicifugae Rhizoma extract attenuates oxidative stress via the regulation of NRF2 [9,19]. These have shown that promoting NRF2 activity with some beneficial drugs can protect organs from oxidative damage. Therefore, we can speculate that Se supplements can also be regulated KEAP1/NRF2 pathway.

Under normal conditions, NRF2 and KEAP1 bind to each other in the cytoplasm. Only when ROS is produced, NRF2 accumulates in the nucleus and regulates downstream proteins [47]. Therefore, when NRF2 enters the nucleus, the KEAP1/NRF2 system works. The results of nuclear accumulation of NRF2 suggested that oxidative stress occurred in hepatocytes, a large number of NRF2 accumulated in the nucleus, and Se further increased the accumulation of NRF2. In addition, the results showed that, even without oxidative stress, Se could make a small amount of NRF2 enter the nucleus (Figure 5D,E). Moreover, it was found that the change trend of NRF2 nuclear protein level was the same as that of the expression level of SEPP1 (Figure 5A,D,E). Therefore, we can come to the conclusion that adequate Se can promote the synthesis of SEPP1, so as to regulate the KEAP1/NRF2 pathway to improve oxidative stress.

Then, we also detected the downstream targets of NRF2. The results showed that both the protein level and enzyme activity of GPX1 were decreased significantly in PA-induced AML-12 cells. This indicated that the function of the intracellular antioxidant system was destroyed. However, Se restored the protein level and enzyme activity of GPX1 in PA-induced AML-12 cells (Figure 5F,G). Adequate and appropriate Se could restore the cell viability of H_2_O_2_-induced AML-12 cells and the protein level of NQO1, a key downstream protein of NRF2 (Figure 6C). These results suggest that, in the liver, Se can promote SEPP1 synthesis to regulate the KEAP1/NRF2 pathway, so as to regulate the downstream antioxidant enzymes of NRF2 and maintain the normal operation of the intracellular antioxidant system.

In summary, all of the results show that Se can reduce liver injury by inhibiting lipid accumulation and downregulate the expression of lipogenic genes to prevent the “first hit” of NAFLD. Meanwhile, Se can regulate the KEAP1/NRF2 pathway to defense against hepatocyte oxidative stress, so as to resist the “second hit”.

## 5. Conclusions

Dietary selenium reduces hepatic injury through inhibiting liver lipid accumulation in HFD-elicited obese mice. In addition, Se supplement increases the expression of SEPP1, thereby activating the KEAP1/NRF2 pathway to protect hepatocytes from oxidative stress.

## Figures and Tables

**Table 1:** Primers for real-time PCR amplifications.

Gene	Gene NCBI ID	Forward Primer	Reverse Primer
Mus *Acc*	1299249	CCGAGAAGCAGAAACACGACG	CTACCACATCAAGGCTCCGAAT
Mus *Fasn*	7988	ATGGATGAGACCTCCCCAAG	AGAGCTTCTTAAGTAGAGAC
Mus *β-actin*	10455	ACAGAGCCTCGCCTTTGCCGA	CATGCCCACCATCACGCCCTGG
Mus *Srebp1*	1313979	ACCCTGGAGGACACGCTGCTAG	GCTTCTGCAAACCTGCGGGAAAC
Mus *Sepp1*	10016	CTGACATTAAGGTGGTTGAC	CACAGCAATCTTCGGTTATG

## Data Availability

Data is contained within the article and supplementary materials.

## Supplementary Materials

The following supporting information can be downloaded at: https://www.mdpi.com/article/10.3390/antiox11020349/s1, Figure S1: The viability analysis of of AML-12 cells treated with different concentrations of Na_2_SeO_3_, L-SeMC and PA; Figure S2: The viability analysis of of AML-12 cells treated with different concentrations H_2_O_2_.
