# Supplementary material for: Dietary Selenium Alleviated Mouse Liver Oxidative Stress and NAFLD Induced by Obesity by Regulating the KEAP1/NRF2 Pathway
Source: Antioxidants (Basel). 2022 Feb 10;11(2):349. doi: 10.3390/antiox11020349 (PMC8868436; doi:10.3390/antiox11020349)
Supplement: Supplementary file 1 [file antioxidants-11-00349-s001.zip › antioxidants-1560226-supplementary.pdf]

Supplementary Materials for

Dietary selenium alleviated mouse liver oxidative stress and NAFLD induced by obesity by regulating Keap1/Nrf2 pathway

Yi Wang [1], BingBing Liu [1], Peixuan Wu [2], Yi Chu [1], SiSi Gui [1], Yazhen Zheng [1],

Xiaodong Chen [1,*]

Correspondence: chenxd@mail.hzau.edu.cn; Tel.: +86 27 87282091

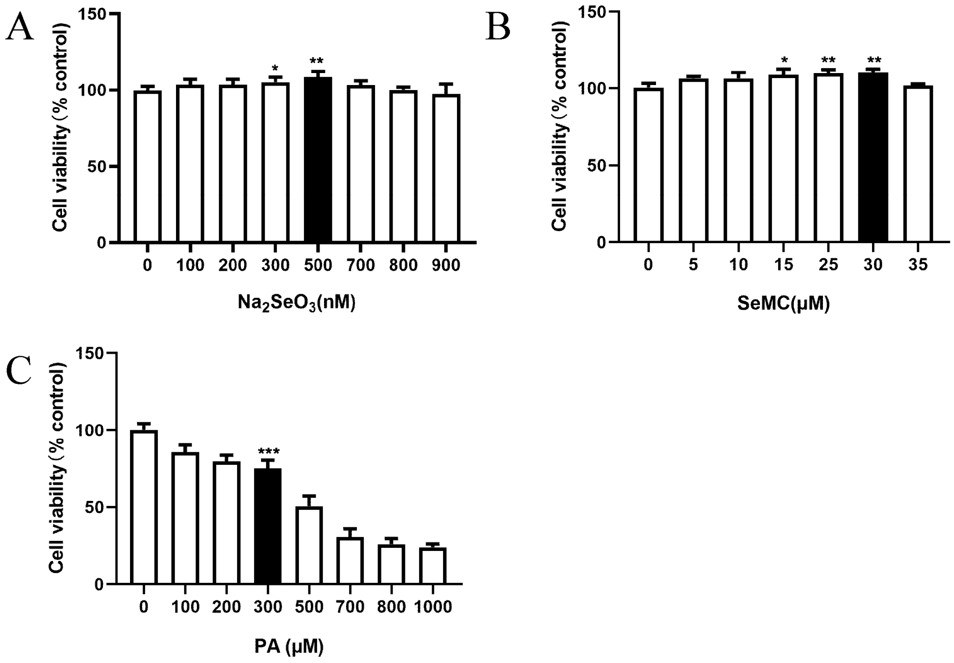

Supplementary Figure S1. (**A**) Cell viability of AML-12 cells was measured by CCK-8 after being treated with different concentrations of Na_2SeO_3 (0-900 nM) for 24h (n = 7, **p < 0.01, *p < 0.05). (**B**) Cell viability of AML-12 cells was measured by CCK-8 after being treated with different concentrations of L-SeMC (0-35μM) for 24h (n = 7, **p < 0.01, *p < 0.05). (**C**) Cell viability of AML-12 cells was measured by CCK-8 after being treated with different concentrations of PA (0-1000μM) for 24h (n = 7, *** p < 0.001).

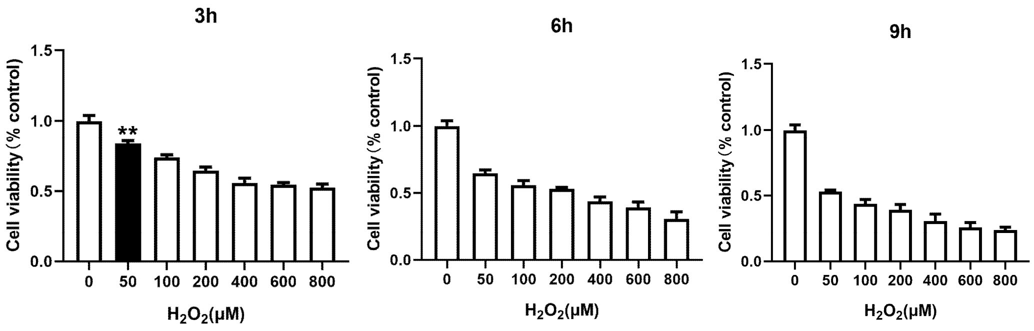

Supplementary Figure S2. Cell viability of AML-12 cells was measured by CCK-8 after being treated with different concentrations of H_2O_2 (0-800μM) for 3h, 6h, 9h (n = 7, **p < 0.01, *p < 0.05).
